# AuNis modified AlGaN/GaN HEMT biosensor for reliable detection of small Rho GTPases in Jurkat T cell lysate

**DOI:** 10.1038/s41598-025-18072-0

**Published:** 2025-10-06

**Authors:** Najihah Fauzi, Chien Fung Chong, Amirul Firdaus, Hiroshi Kawarada, Ana Masara Ahmad Mokhtar, Shaili Falina, Mohd Syamsul

**Affiliations:** 1 Institute of Nano Optoelectronics Research and Technology (INOR), Universiti Sains Malaysia, Sains@USM, 11900 Bayan Lepas, Pulau Pinang Malaysia; 2 Bioprocess Technology Division, School of Industrial Technology, Universiti Sains Malaysia, 11800 Gelugor, Penang, Malaysia; 3 Faculty of Science and Engineering and Institute of Nano Science and Nano Engineering, Waseda University, Shinjuku, Tokyo 169-8555 Japan; 4 Power Diamond Systems Inc, Shinjuku, Tokyo 169-0051 Japan; 5 Collaborative Microelectronic Design Excellence Center (CEDEC), Universiti Sains Malaysia, Sains@USM, 11900 Pulau Pinang, Bayan Lepas, Malaysia

**Keywords:** Cancer, Biomarkers, Diseases, Engineering, Nanoscience and technology, Physics

## Abstract

A highly sensitive and stable biosensor was crucial for the early screening and treatment of diseases. As one of the most promising and powerful platforms, AlGaN/GaN HEMT biosensors have enormous potential owing to their distinctive 2DEG channel features. While gold nanomaterials offer great potential for creating highly sensitive sensing surfaces due to their nanoscale size, their lack of robustness and inconsistent concentration on the HEMT surface hinders the reliability and reproducibility of biosensors. Herein, gold nanoislands (AuNis) are utilized for the first time as a sensing membrane on AlGaN/GaN HEMT devices for biosensing applications. The high sensitivity for pH detection verified the excellent AuNis sensing capability. For biosensing applications, glutathione S-transferase–p21-activated kinase1–GTPase-binding domain (GST-PAK1-GBD; residues 56–272) served as a bioreceptor for small Rho GTPases in Jurkat T-cell lysate detection, achieving high current and voltage sensitivities of 9.10% and 33.00% at 3 × 10⁻⁷ g/mL, respectively. Benefitting from the large surface binding site of the AuNis sensing surface, a wider range of Jurkat T-cell lysate detection from 3 × 10^−16^ to 3 × 10^−7^ g/mL and an ultra-low limit of detection of 3 × 10^−16^ g/mL are obtained. Furthermore, excellent reproducibility with a reported correlation coefficient (R^2^ ≥ 0.950) across multiple sensors and > 98% signal recovery after regeneration cycles further validates the sensor’s performance and reusability. The modification of the sensing surface with AuNis marks a significant advancement toward the development of robust and reproducible biosensors.

## Introduction

Small Rho GTPases from the Rho family, such as Rac and Cdc42, play key roles in regulating actin cytoskeleton changes and cell morphology, which control the cell’s growth, survival, and migration. Mutations or alterations of Rac and Cdc42 in their guanosine triphosphate (GTP)-bound active form can contribute to cancer development^1^. Therefore, detecting small Rho GTPases in cancer cells is crucial for identifying dysregulated signaling pathways that promote cancer progression^2^. One of the primary effectors downstream of these small Rho GTPases is p21-activated kinase 1 (PAK1), a serine/threonine kinase increasingly recognized for its role in cancer pathogenesis^3^. PAK1 has emerged as a critical player in cancer biology, being implicated in nearly all stages of tumor development. Its overexpression has been observed in several cancers, including ovarian, breast, and leukemia, highlighting its potential as both a therapeutic target and diagnostic biomarker^4–6^. PAK1 is activated upon binding to the GTP-bound forms of Rac1 and Cdc42, which act as molecular switches to initiate downstream signaling^7^. Once activated, PAK1 promotes oncogenic pathways, accelerates cell cycle progression, and drives cytoskeletal reorganization, collectively contributing to cancer progression^5^. In this study, the activation state of small Rho GTPases is detected using an effector pull-down assay, which utilizes the GTPase-binding domain (GBD) as a bioreceptor^8^. This GBD domain acts as an effective molecular bait, specifically capturing and purifying active, GTP-bound proteins from Jurkat T-cell lysate^9^. Jurkat T-cell, an immortalized T-lymphocyte cell line, are commonly used in studies related to acute T-cell leukemia and serve as a cell surface biomarker to diagnose leukemia for early detection^10^. This GBD domain exhibits high specificity toward the GTP-bound forms of Rac1 and Cdc42, thereby ensuring selective interaction within the Rho/PAK1 signaling cascade^11^. Utilizing this specific molecular recognition significantly enhances detection sensitivity, allowing even a small number of target cells or proteins to be captured due to the strong affinity between the GST-PAK1-GBD and activated small Rho GTPases in Jurkat T-cell lysate.

Field-effect transistor (FET)-based biosensors are a type of surface affinity sensor that operates by utilizing the molecular recognition properties of immobilized receptors to selectively detect biological targets present in a sample solution. When the target molecule binds to the receptor on the sensor surface, it induces a change in the electrical properties of the transistor, such as charge distribution or surface potential, leading to a measurable electrical signal^12^. In recent years, AlGaN/GaN high electron mobility transistors (HEMTs) have shown great potential applications among various types of FET-based biosensors, owing to their unique properties of two-dimensional electron gas (2DEG) channel induced by spontaneous and piezoelectric polarization between AlGaN/GaN heterostructures^13^. Since the 2DEG is located near the AlGaN surface, it is highly responsive to minor changes at the sensing interface, enabling sensitive detection of biological events and pH variations^14^. Furthermore, the GaN material exhibits chemical inertness and high-temperature endurance, making the AlGaN/GaN-based sensor suitable for operating under harsh environments, including acidic and alkaline solutions^15^. In addition, the surface functionalization plays an important role in enhancing the detection of biosensors.

Nanomaterials have emerged as promising candidates for the development of highly sensitive biosensors due to their extremely small size. Among them, gold (Au) nanostructures stand out as an excellent choice, playing a critical role as a sensing membrane modifier and a linker in biosensor fabrication. Gold effectively bridges the interface between the inorganic sensor platform and biological receptors, facilitating efficient signal transduction. Hence, gold is widely employed as an immobilization platform, an electrocatalyst, and an electron migration enhancer, all of which contribute to improving the sensitivity and stability of biosensor performance. A key benefit of gold is its ability to be easily functionalized with biomolecules while preserving the activity of biomolecules^16^. Gold nanoparticles (AuNPs), in particular, are commonly used in HEMT-based biosensors^17^. However, conventional AuNPs-based surface modification techniques are not particularly robust, which may compromise biosensor reliability. Surface functionalization of the HEMT sensing area is typically required prior to AuNPs deposition to prevent nanoparticle detachment, adding complexity to the fabrication process. Additionally, achieving consistent reproducibility remains challenging due to aggregation during AuNPs immobilization, leading to variability in nanoparticle density on the sensing surface. To overcome these limitations, more efficient and simplified fabrication strategies are needed to achieve better control over nanoparticle size, morphology, and surface distribution, thereby improving biosensor performance and reproducibility.

In this paper, the AuNis have been developed as a sensing membrane on the electrolyte gate-HEMT biosensor for pH and Jurkat T-cell lysate detection. The thickness of the Au film used to produce AuNis is 2 nm, offering a cost-effective sensor, followed by a brief annealing process to form the AuNis, which involves simple process fabrication. The distinctive structure of islands provides more surface-active sites and high adsorption capacity for hydrogen ions (H^+^) and hydroxide ions (OH^−^) for pH detection. Highly sensitive and stable pH sensing results as a proof-of-concept confirm the biosensing ability of the AuNis HEMT. To the best of our knowledge, this study is the first to report a biosensing application that targets the Rho/PAK1 signaling pathway using an AuNis-functionalized AlGaN/GaN HEMT biosensor. Glutathione (GSH) was employed as a molecular linker to immobilize GST-PAK1-GBD (residues 56–272) on the sensor surface, enabling the selective detection of activated small Rho GTPases in Jurkat T-cell lysate. The biosensor demonstrated a broad linear detection range from 3 × 10⁻^16^ to 3 × 10⁻⁷ g/mL, with an ultra-low detection limit of 3 × 10⁻^16^ g/mL. Furthermore, the consistency of the measurement and the negligible current difference after the washing step prove the reproducibility and the regeneration of the AuNis HEMT biosensor, respectively.

## Materials and methods

### Optimization formation of AuNis

The initial optimization of AuNis formation was carried out by depositing Au films with different thicknesses (2 and 3 nm) on a Si substrate using e-beam evaporation, followed by annealing at 400 °C for 60 sec in N_2_ via rapid thermal processing (RTP). The surface morphology of AuNis formation was evaluated by field emission scanning electron microscopy (FESEM).

### AuNis HEMT sensor fabrication

**Fig. 1:**
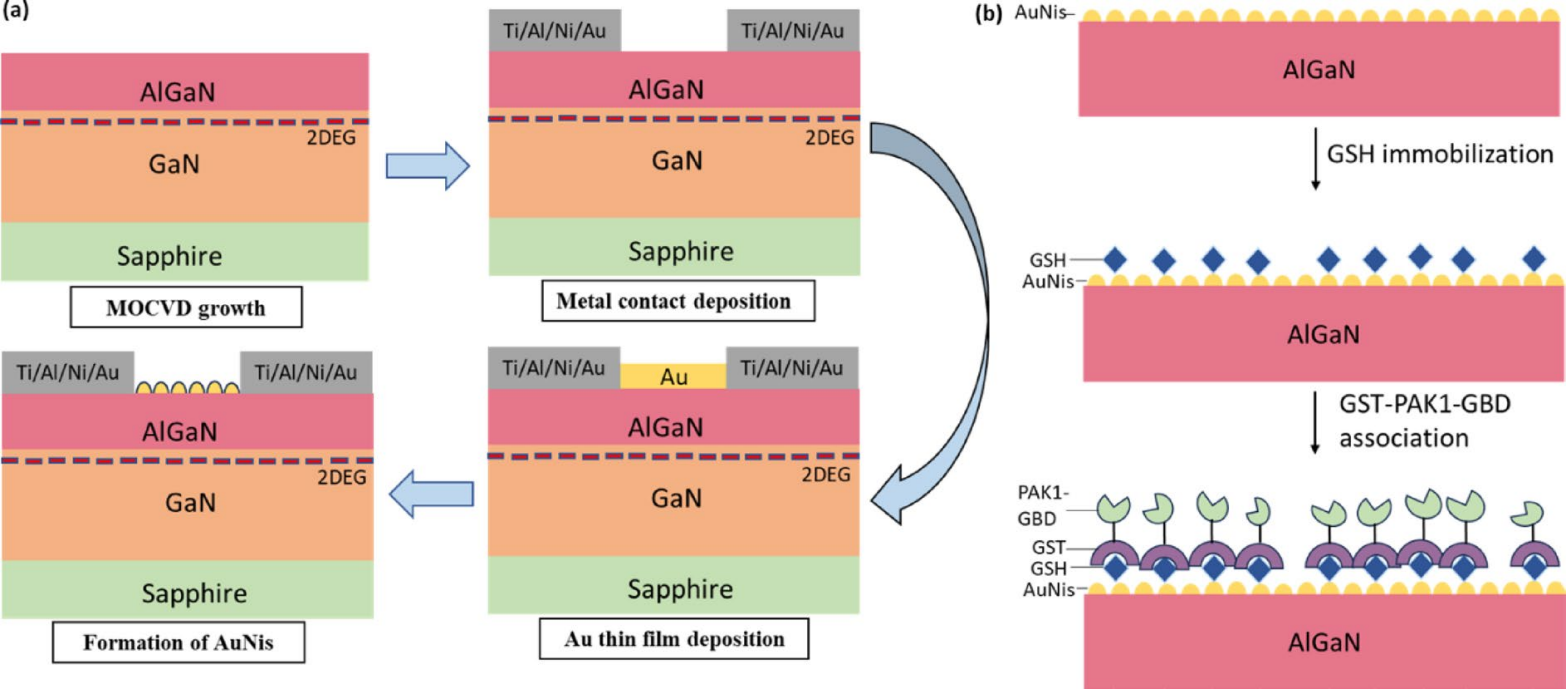
The schematic diagram process of (**a**) AuNis HEMT sensor fabrication and (**b**) the surface functionalization immobilization on the AuNis HEMT sensing surface, respectively.

The AlGaN/GaN were grown on a sapphire substrate by metal organic chemical vapor deposition (MOCVD) in a horizontal flow Taiyo Nippon Sanso Corporation (SR4000KS-HT) reactor. The AlGaN/GaN HEMT heterostructures were fabricated with the GaN layer two-step growth method, which is composed of a 20 nm GaN buffer layer, a 1.2 µm-thick first GaN layer followed by a 2.2 µm-thick second GaN layer, and a 22 nm AlGaN layer with 25% Al content. Then, the size of the device was made into 5 × 5 mm in length and width. The ohmic contact was created with source and drain metallization layers using electron beam metal deposition and made of Ti/Al/Ni/Au with thicknesses of (20 nm/160 nm/55 nm/45 nm). Following that, the device was thermally treated with rapid thermal annealing (RTP) at 860 °C for around 60 sec with nitrogen gas (N_2_). Then, the exposed (2 × 5 mm) area between the source and drain region was selectively deposited with Au film. A thin Au film with a 2 nm thickness was deposited by electron beam metal deposition. Finally, the AuNis sensing area was formed after being thermally treated for approximately 60 sec at 400 °C in ambient N_2_ using RTP. Figure 1a illustrates the schematic diagrams of the AuNis HEMT sensor fabrication process. The surface morphology of the AuNis was investigated by FESEM and atomic surface microscopy (AFM) in the tapping mode (Nanoscope IV/Multimode, Digital Instruments, United States). Then, the fabricated device was mounted on a glass substrate. The connectivity of the sensor was made with two wires that were attached to the source and drain using conductive and non-conductive epoxy, sequentially. On that account, the sensor was eventually encapsulated in epoxy to prevent any leakage current, keeping only the sensing area open for the next biofunctionalization process.

### GST-PAK1-GBD expression

A glutathione-S-transferase (GST)-fusion protein construct of pGEX-2TK-PAK1 G-protein binding domain (GBD; residue 56–272) was kindly provided by Dr. Darerca Owen (University of Cambridge, UK). Expression of this construct was performed in *Escherichia coli* BL21 for 5 h at 37 °C after induction with 0.1 mM isopropyl β-D-1-thiogalactopyranoside (IPTG). Following expression, cells were pelleted and resuspended in ice-chilled buffer (150 mM NaCl, 16 mM Na_2_HPO_4_, 4 mM NaH_2_PO_4_) containing 20 μg/mL phenylmethylsulfonyl fluoride (PMSF) protease inhibitor (E-EL-SR002, Elabscience®). Cell suspension was then sonicated using an Omni Sonic Ruptor 400 Ultrasonic Homogenizer in three 5-min cycles at 50% amplitude, with samples placed on ice for 5 min between each cycle to prevent overheating. Subsequently, the lysates were centrifuged, and the resulting supernatants were affinity purified using GST focurose 4FF (E-CM-AF04, Elabscience®), concentrated using an Amicon® Ultra-15 centrifugal filter, and quantified by A_280_ measurement.

### Surface functionalization immobilization

Firstly, glutathione (GSH) Focurose 4FF (E-CM-AF04, Elabscience®), a highly cross-linked 4% agarose, was centrifuged at 6000 ×*g* for 5 min and washed multiple times with 1 × phosphate-buffered saline (PBS, pH 7.4) to equilibrate the beads. Subsequently, 10 μL of 5 mg/mL equilibrated GSH was dropped onto the AuNis sensing surface using a micropipette (1–10 μL range) and incubated at 4 °C for 24 h to allow the formation of a self-assembled monolayer (SAM). After incubation, the sensor was rinsed with 1 × PBS for 10 s and dried using nitrogen (N₂) gas. Following this, 10 μL of 10 mg/mL GST-PAK1-GBD (residues 56–272) was pipetted onto the GSH-functionalized AuNis surface and incubated at 4 °C for 1 h to serve as a specific bioreceptor for small Rho GTPases from Jurkat T-cell lysate detection. To minimize non-specific binding, the sensor was immersed in a 6 M bovine serum albumin (BSA) solution for 5 min at 4 °C, followed by two brief rinses in 1 × PBS (10 s each) to remove any unbound BSA. Figure 1b illustrates the surface functionalization immobilization process on the AuNis HEMT sensing surface.

### pH and Jurkat T-cell lysate preparation

A 1 × PBS solution with an initial pH of 7.4 was adjusted to the desired pH levels by the incremental addition of 0.1 mol/L hydrochloric acid (HCl) for acidic solutions or 0.1 mol/L potassium hydroxide (KOH) for basic solutions. The solutions were continuously stirred, and the pH was monitored in real time using the pH meter (Hanna instruments) until the target pH value was reached. This procedure was repeated for all required pH values.

Jurkat T Clone E6-1 (ATCC Cat. No. TIB-152™), a human acute leukemia T-cell line, was cultured in Roswell Park Memorial Institute (RPMI)-1640. The cell lines were maintained in a controlled environment, a humified incubator with 5% CO_2_ at 37 °C, while cells used for experiments were between passages 5 to 20 to minimize phenotypic drift. Upon reaching ~ 80–90% cell confluency, the cells were subjected to lysis using a buffer containing 20 mM Tris–HCl (pH 7.5), 150 mM NaCl, 1 mM dithiothreitol (DTT), 5 mM MgCl_2_, 5 mM β-glycerophosphate disodium salt hydrate, and 0.5% NP-40 alternative, after which the supernatant was collected by centrifugation and protein quantification was performed using a BCA protein colourimetric assay (E-BC-K318-M, Elabscience®).

### Biosensor measurement

**Fig. 2:**
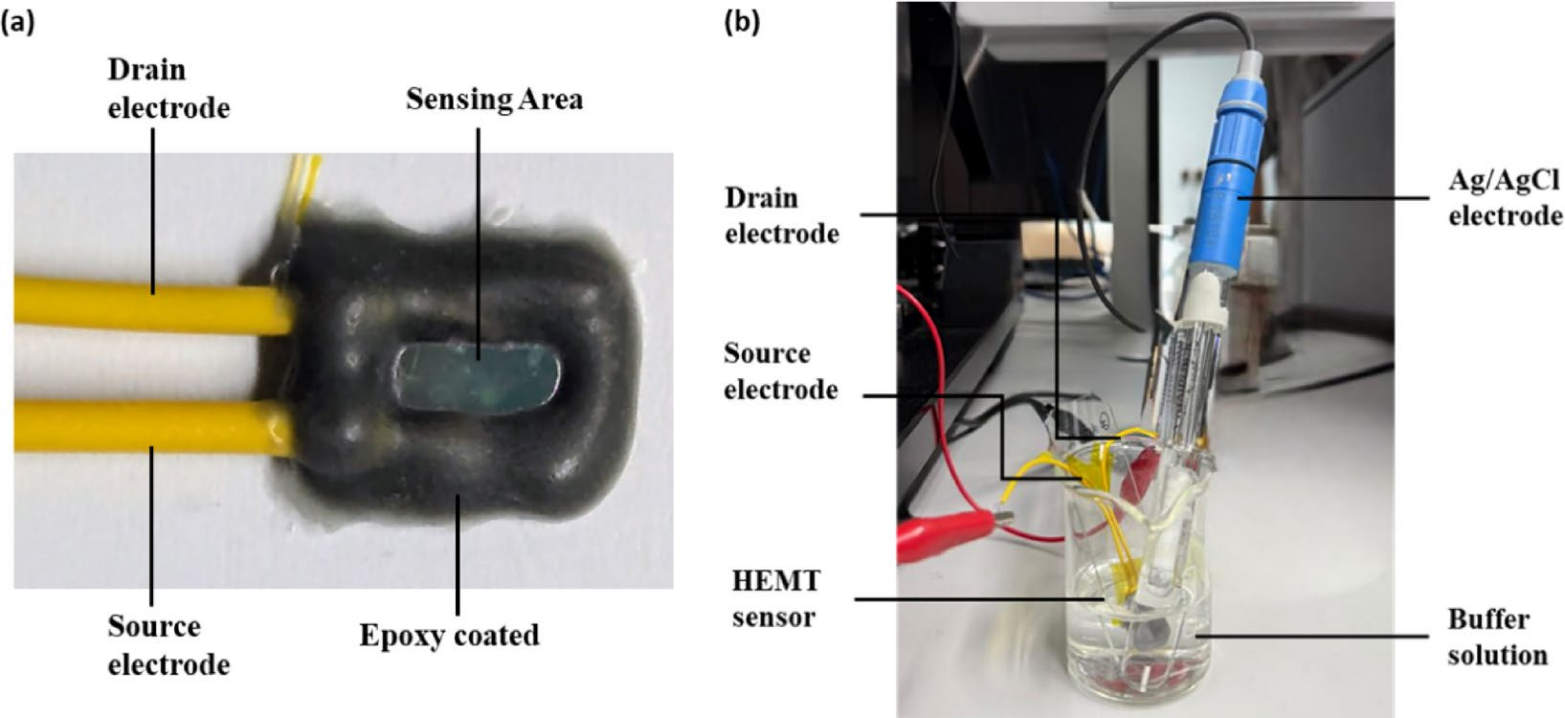
The image of (**a**) fabricated HEMT device and (**b**) the testing setup for sensor measurement.

Electrical measurements were carried out by fully immersing both the sensor and a reference electrode in adequate buffer solutions, using a Source Measuring Analyser (Keithley 2400 Source Meter®). The electrical properties of the biosensor were evaluated after each step of the surface functionalization process. Subsequently, the biosensor was individually exposed to Jurkat T-cell lysate concentrations ranging from 3 × 10⁻^16^ g/mL to 3 × 10⁻⁷ g/mL for 10 min prior to measurement. The output (I_DS_–V_DS_) and transfer (I_DS_–V_GS_) characteristics were recorded at a constant bias V_GS_ of − 1 V and a constant V_DS_ of 1 V, respectively, under varying pH conditions and Jurkat T-cell lysate concentrations. The gate-to-source voltage (V_GS_) of − 1 V was selected over other negative voltages based on optimal sensor performance for this specific design. Time-dependent measurement was then performed by monitoring I_DS_ at a constant V_DS_ = 0.5 V for 60 sec. Continuous monitoring can raise device temperature due to prolonged biasing, inducing self-heating that increases gate leakage from the 2DEG channel and causes baseline shifting in the HEMT. These effects can compromise the stability and reliability of the sensor. Therefore, a pulsed gate bias operation with a short duration of 1 s and a gate voltage of − 1 V was employed in this study. This technique has been extensively validated in previous studies, such as by Tai and research groups, where pulsed operation in HEMT-based sensors significantly suppressed heat-induced effects, thereby enabling stable and reliable sensor output^18^. To minimize device-to-device variability, the sensing response was defined as the change in drain current (ΔI_DS_ =|I_DS_—I_0_|), rather than the absolute drain current^19^. This approach was similarly applied to voltage sensitivity, using the change in gate voltage (ΔV_GS_). All measurements were conducted at room temperature. Figure 2a, b display the image of the fabricated HEMT device and the testing setup used for sensor measurements, respectively.

### Protein elution process

After every measurement, the device is washed in 70% ethanol for 5 sec, then immediately immersed in sterile distilled water. The process is repeated 5 times to eliminate any contaminant on the sensor. Following that, the sensor was immersed in 6 M guanidine hydrochloride for 5 sec, briefly rinsed in sterile distilled water, and repeated again 5 times. The elution buffer containing a very high salt concentration disrupts the binding by denaturing the glutathione interaction. Then, the sensor is blown dry with N_2_ before using the device for subsequent detection.

## Results and discussions

### Optimization formation of AuNis

**Fig. 3:**
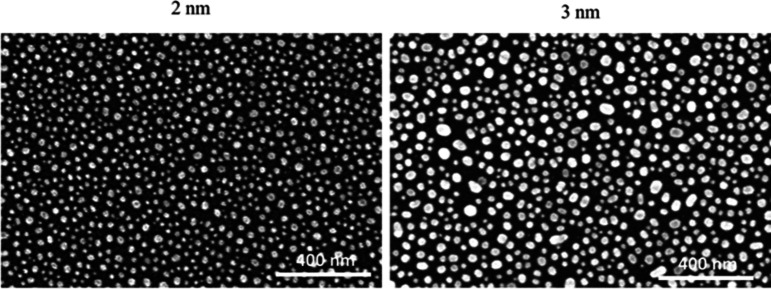
The top view of AuNis FESEM image on Si substrate with Au thickness of 2 nm and 3 nm.

Figure 3 shows the FESEM analysis of gold (Au) films with different thicknesses deposited on a silicon (Si) substrate to optimize the formation of gold nanoislands (AuNis). The 2 nm Au film produced smaller and more uniformly distributed nanoislands, with sizes ranging from 7 to 12 nm. In contrast, the 3 nm film resulted in larger, less uniform nanoislands with a broader size distribution of 6 to 24 nm. The improved uniformity and reduced size of the AuNis from the 2 nm film contribute to a higher surface-area-to-volume ratio, which is beneficial for biosensing applications. This is due to the fact that biological responses to nanoparticles are closely influenced by their surface-area-to-volume ratio. Nanoparticles with smaller sizes offer a larger surface area relative to their volume, enabling more protein adsorption from the surrounding environment and promoting enhanced biomolecular interactions^20^.

### Surface characterization of AuNis HEMT

**Fig. 4:**
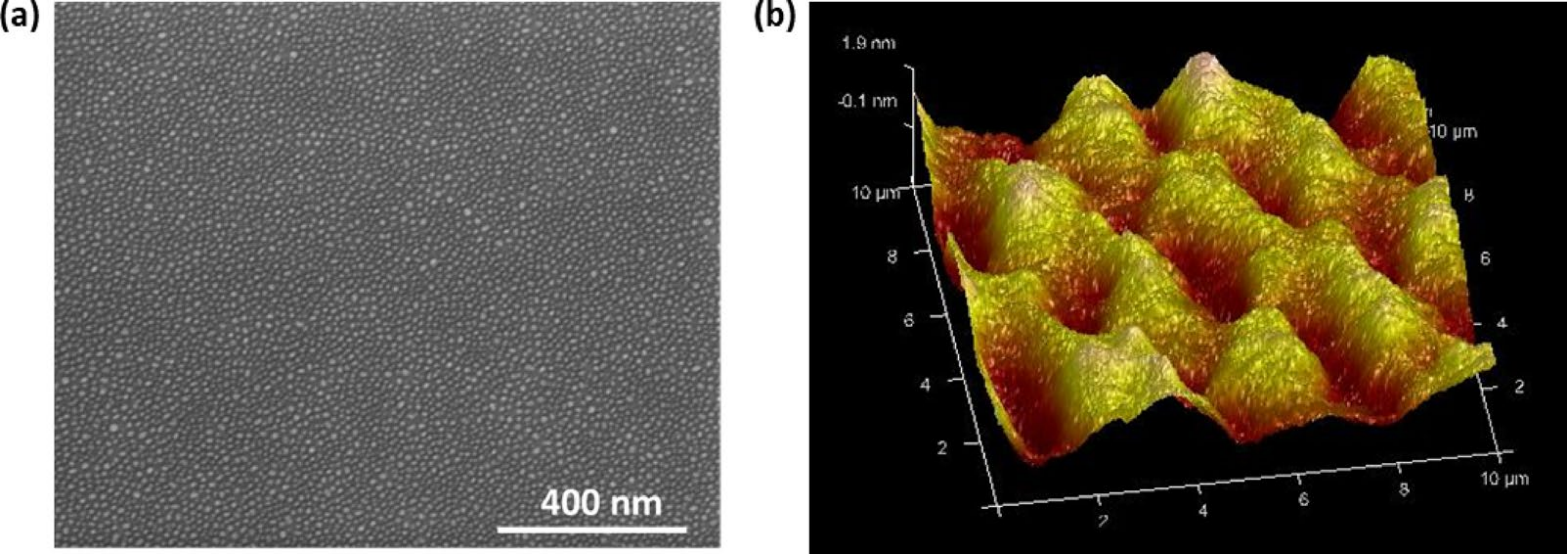
The images of AuNis on the HEMT surface by (**a**) FESEM and (**b**) AFM analysis.

The surface morphology of the AuNis has been studied by FESEM and AFM analysis. Figure 4a displays the top view of the AuNis FESEM image showing uniformly and densely distributed gold nanostructured islands with an average diameter of 7–23 nm. Similarly, the homogenous and smooth surface arrangement of AuNis with a surface roughness of 0.619 nm is shown in Fig. 4b. Notably, the AuNis surface showed no evident cracks or crystalline grains after annealing. Contrary to AuNPs, which easily aggregated and exfoliated from the surface after complete fabrication and drying^21^. Further, the isolation of AuNis provides a large surface area-to-volume ratio and more binding sites for pH and small Rho-GTPases from Jurkat T-cell lysate detection.

### Sensing mechanism of the HEMT biosensor

The sensitivity of the HEMT biosensor relies on 2DEG concentration near the sensing surface. Any changes in surface charge state (positive or negative) will alter surface potential and modify the 2DEG density at the AlGaN/GaN interface. When gate voltage (V_g_) is applied to the gate electrode, an electric double layer (EDL) forms at the interface between the biosensor’s sensing surface, the gate electrode, and the sample solution. This generates a capacitance across the solution (C_s_), which controls the voltage drop in the HEMT biosensor (V_d_). This means that when C_s_ changes, the V_d_ changes, resulting in changes in HEMT drain current (I_d_). The C_s_ can be varied under different conditions, including when there are changes in surface charge state due to the functionalization of the sensing surface, different ionic strength mediums, and electrostatic interaction via receptor-ligand binding^18^. Since the V_g_ applying on the HEMT biosensor remains constant for any pH and Jurkat T-cell lysate concentration, the changes in I_d_ are directly related to the changes in potential drops across the sample solution (V_s_) solely resulting from the pH variation and receptor-ligand binding^22^. The V_g_ applied can be represented mathematically as

1 $${\text{V}}_{{\text{g}}} { = }\Delta {\text{V}}_{{\text{s}}} { + }\Delta {\text{V}}_{{\text{d}}}$$

The pH sensing mechanism is governed by the adsorption and dissociation of H⁺ ions on the sensing membrane, resulting in the formation of positively or negatively charged sites. Au, with its distinctive electronic configuration consisting of a filled 5d orbital and a partially filled 6s orbital, facilitates efficient charge transfer with H⁺ and OH⁻ ions^23,24^. This charge interaction not only stabilizes ion adsorption but also induces significant shifts in the interfacial potential, thereby establishing the fundamental basis underlying pH detection^25^.

### pH detection

**Fig. 5:**
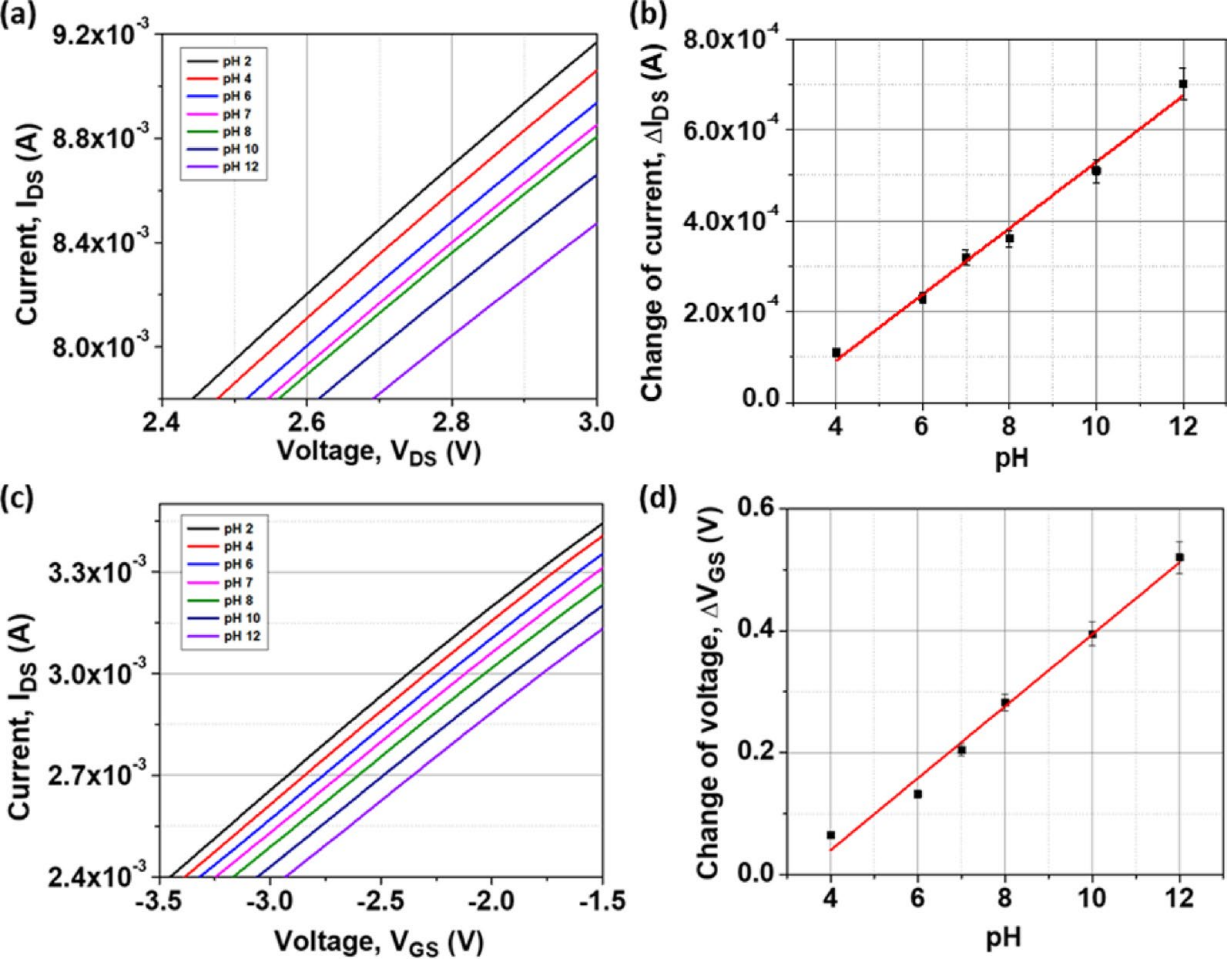
The graph of (**a**) the I_DS_-V_DS_ curve, (**b**) the current sensitivity and linearity, (**c**) the I_DS_-V_GS_ curve, and (**d**) the voltage sensitivity and linearity with varying pH values.

pH detection can be an effective approach to evaluate the performance of AuNis as a sensing surface on AlGaN/GaN HEMT biosensors. The output current response (I_DS_-V_DS_) curves are depicted in Fig. 5a measured at a constant V_GS_ of − 1 V in pH 2 to pH 12 solutions. The I_DS_ values decreased as the pH increased from pH 2 to pH 12. This is because, at higher pH values, OH⁻ ions have a greater concentration than H⁺ ions, leading to their dominant adsorption on the Au sensing membrane. The resulting accumulation of negative charges (OH⁻ ions) lowers the 2DEG concentration and, consequently, reduces the response current. Likewise, Pyo et al. demonstrated a similar finding in which a lower H^+^ concentration in the pH buffer solution leads to a decrease in current^26^. The pH sensitivity and linearity of the device were defined by the linear relationship between the ΔI_DS_ and pH at a V_DS_ = 3 V as shown in Fig. 5b. Here, the pH 2 current was referred to as a baseline current, which determined the current sensitivity of 72.78 μA/pH with the linearity of R^2^ approximately at 0.988. The transfer curve of drain current (I_DS_) with the function of gate voltage (V_GS_) of the AuNis HEMT pH sensor is shown in Fig. 5c at a constant drain voltage of V_DS_ = 1 V. The transfer curve shifted to the right from pH 2 to pH 12 with an increasing value of threshold voltage (V_TH_) and a contrary decrease in I_DS_. Then, the changes value of ΔV_GS_ at a drain current of 3 mA as a function of pH were plotted in Fig. 5d. The voltage sensitivity was found at 58.94 mV/pH with a linearity of R^2^ = 0.986. This proved that the modulation of the 2DEG concentration impacts the performance of the AuNis HEMT sensor^27^. Hence, the high current and voltage sensitivity and good linearity in acidic and basic solutions suggest that AuNis provide more surface binding sites to improve the performance of the AlGaN/GaN HEMT biosensor. Further maximizing detection sensitivity in biosensing, the choice of biomarker and its immobilization method should be considered.

### Surface functionalization immobilization

**Fig. 6:**
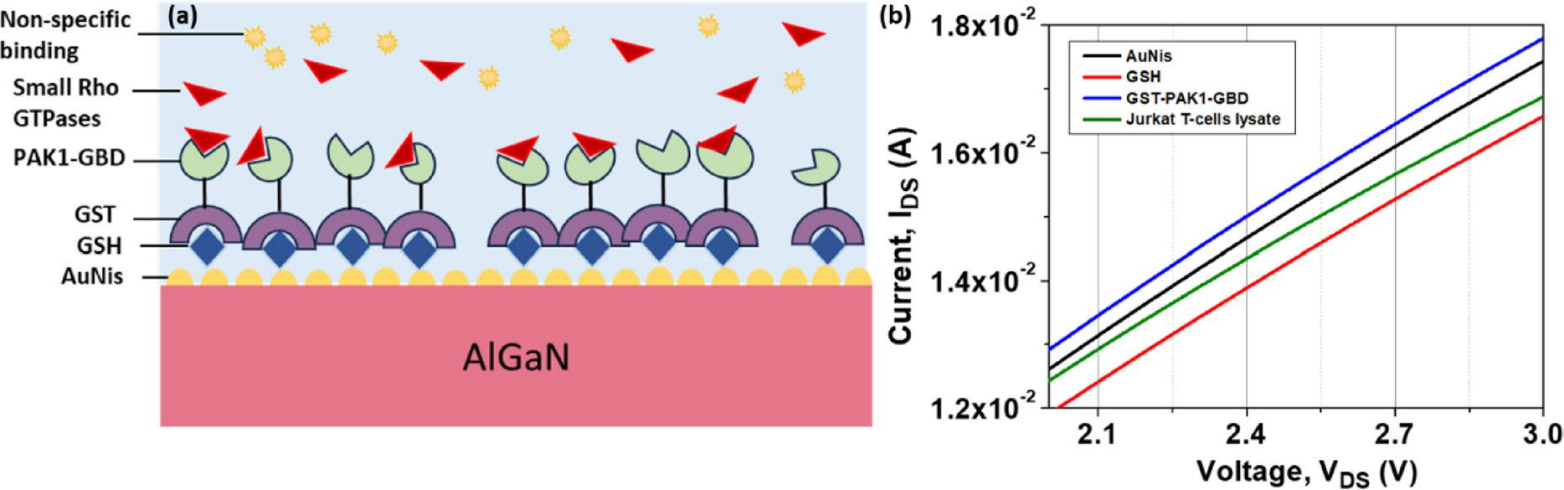
The surface modification of the AuNis HEMT biosensor with (**a**) a schematic diagram and (**b**) the I_DS_–V_DS_ characteristic.

Since AuNis as a sensing surface of AlGaN/GaN HEMT biosensor has proven to be highly sensitive in pH solutions, further explore the feasibility of biomolecular sensing. Here, the Jurkat T-cell lysate was selected as the target for detection due to its characteristic deregulation of the Rho/Pak1 signaling pathway. Glutathione (GSH) was utilized as a linker molecule, forming strong Au–S bonds through the sulfhydryl (–SH) group of its cysteine residue. The formation of the Au–S bond through Au/GSH interaction is well established and has been extensively characterized in previous studies^28–30^. Subsequently, GST-PAK1-GBD was immobilized onto the GSH layer via the high-affinity interaction between the glutathione-binding site (G-site) of GST, located in its N-terminal domain^31,32^. The GST-PAK1-GBD construct acts as a receptor, capturing activated GTP-bound small Rho GTPases from Jurkat T-cell lysate through the C-terminal domain of PAK1, as illustrated in Fig. 6a^33^. The successful immobilization of the functional layers was confirmed by monitoring changes in the drain current (ΔI_DS_) from the I_DS_–V_DS_ characteristics of the AuNi_S_ HEMT biosensor, as shown in Fig. 6b. The sensing characteristics of the electrolyte-gated HEMT biosensor were evaluated based on the detection of proteins through their net surface charges upon successful binding to the AuNis sensing surface^34^. A decrease in drain current was observed following the immobilization of GSH on the AuNis surface. This is attributed to the formation of Au–S bonds between gold and the sulfhydryl (–SH) group of the cysteine residue in GSH, leaving a net negative charge composed of an NH₃⁺ and a COO⁻ group from the glutamate residue and a COO⁻ group from the glycine residue^35,36^. The accumulation of negative charges at the sensing surface repels electrons from the 2DEG channel near the AlGaN interface, inducing charge redistribution in the electrical double layer (EDL), altering the capacitance across the solution, and leading to a reduction in drain current^37^. Subsequently, a significant increase in current was observed after exposure to GST-PAK1-GBD, attributed to the net positive charge predominantly contributed by the amine groups of the PAK1 (residues 56–272) domain. The positive charge on the surface enhances electron accumulation in the 2DEG channel, resulting in an increased drain current. Lastly, negative charges are introduced onto the sensing surface when GST-PAK1-GBD binds to small Rho GTPases from the negatively charged Jurkat T-cell lysate^38,39^. This leads to a decrease in the 2DEG concentration, further reducing the drain current.

### Small rho GTPases from Jurkat T-cell lysate detection

**Fig. 7:**
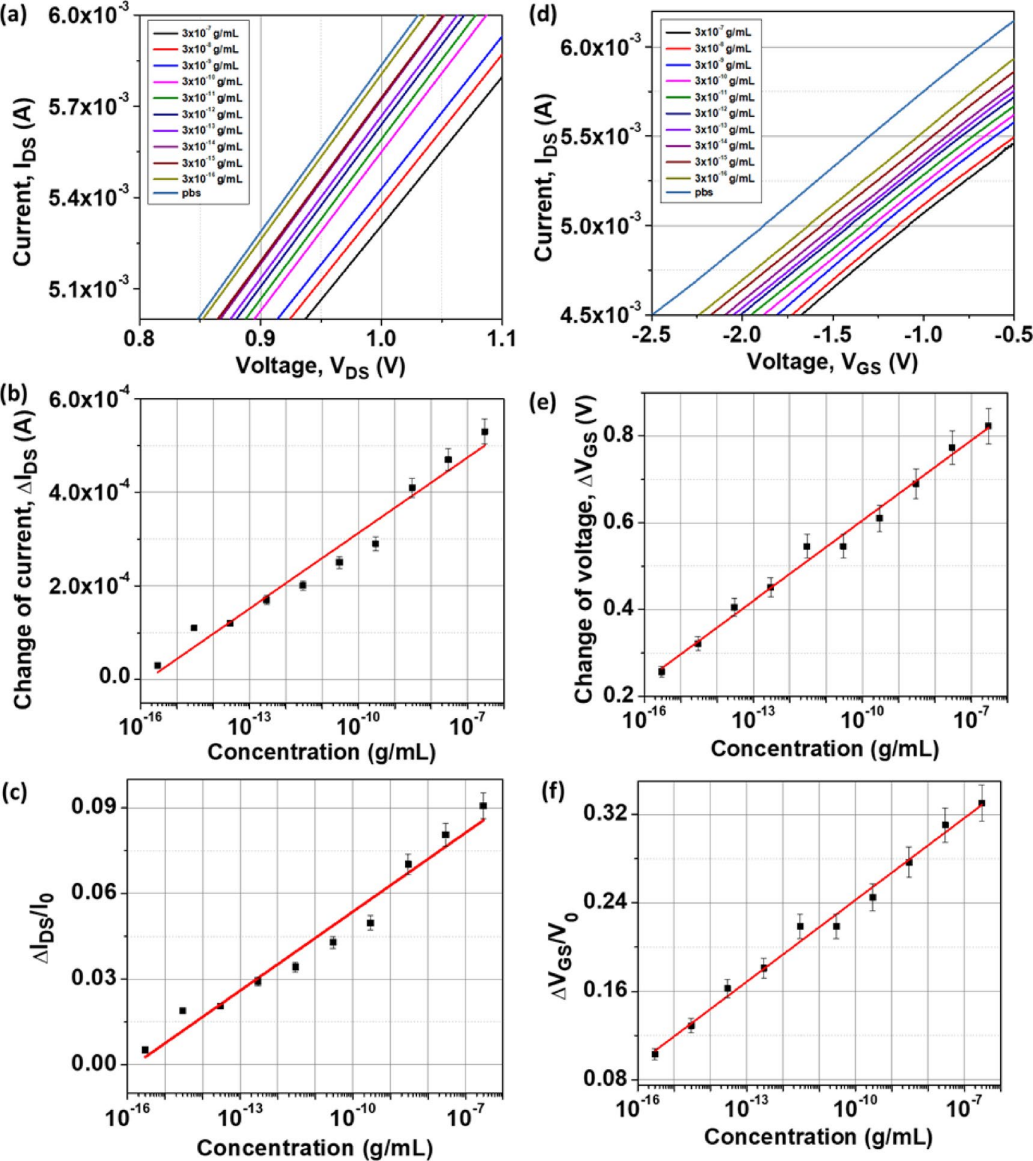
The graph of (**a**) the I_DS_–V_DS_ curve, (**b**) the change of current (ΔI_DS_), (**c**) the current sensitivity and linearity, (**d**) the I_DS_–V_GS_ curve, (**e**) the change of voltage (ΔV_GS_), and (**f**) the voltage sensitivity and linearity with varying Jurkat T-cell lysate concentration.

Figure 7a shows the output characteristics (I_DS_–V_DS_) of the AuNis HEMT biosensor in response to Jurkat T-cell lysate at various concentrations from 3 × 10⁻^16^ g/mL to 3 × 10⁻⁷ g/mL, measured at a constant gate voltage (V_GS_) of − 1 V. The Jurkat T-cell lysate was diluted in 1 × PBS (pH 7.4) to simulate the physiological environment of human serum. The results revealed a decreasing trend in drain current with increasing concentrations of Jurkat T-cell lysate. At higher concentrations, the Jurkat T-cell lysate contains a large amount of small Rho GTPases, which bind more extensively to the GST-PAK1-GBD immobilized on the sensor surface. This interaction introduces a greater density of negative charges onto the sensing region, effectively increasing the negative surface potential. Consequently, the 2DEG channel conductivity of the AuNis HEMT biosensor is further suppressed, leading to a more pronounced decrease in drain current (I_DS_). The drain current shift (ΔI_DS_ =|I_DS_–I_0_|), where I_0_ is the PBS current as a baseline current, was extracted based on the output curves at a fixed V_DS_ value of 1 V. A linear relationship between ΔI_DS_ and log([concentration]) of Jurkat T-cell lysate was displayed in Fig. 7b, presenting good linearity with a correlation coefficient (R) of 0.963. The maximum ΔI_DS_ of 0.53 mA was observed at a Jurkat T-cell lysate concentration of 3 × 10^–16^ g/mL. Specifically, the current shift value was found to be 53.82 μA/decade as the Jurkat T-cell lysate concentration increased tenfold. Sensitivity, a key parameter in evaluating biosensor performance, was calculated as S_I_ =|(ΔI_DS_/I_0_)|× 100%^40^. The sensitivity profile, demonstrating the relationship between ΔI_DS_/I_0_ and the logarithm of Jurkat T-cell lysate concentration, is illustrated in Fig. 7c. The maximum sensitivity reached approximately 9.10% at 3 × 10⁻^7^ g/mL, markedly surpassing the performance of previously reported HEMT biosensors for cancer detection (< 1.0%)^19,41^.

Figure 7d displays the transfer curves (I_DS_ vs. V_GS_) of the AuNis HEMT biosensor measured at a fixed V_DS_ of 1 V for varying concentrations of Jurkat T-cell lysate, ranging from 3 × 10⁻^16^ to 3 × 10⁻⁷ g/mL. As the concentration of the Jurkat T-cell lysate increases, the transfer curve exhibits a progressive positive shift, indicating a change in surface potential due to enhanced biomolecular binding of small Rho GTPases to the GST-PAK1-GBD. The accumulation of negatively charged biomolecules increases the surface charge density, which in turn reduces the electron concentration in the 2DEG channel by electrostatically repelling carriers. This results in a decrease in drain current and a positive shift in the V_TH_, highlighting the sensor’s high sensitivity to surface charge variations induced by target binding. Figure 7e shows that a significant ΔV_GS_ of approximately 823.15 mV was observed at the highest Jurkat T-cell lysate concentration, with a voltage shift rate of 61.60 mV/decade in each concentration. This indicates excellent voltage sensitivity, outperforming previous HEMT-based biosensors with a 11.23 mV/dec potential shift^42^. The response demonstrated strong linearity, with a correlation coefficient (R) of 0.989. The observed voltage shifts are attributed to changes in surface potential resulting from every successful binding of activated small Rho GTPases in the Jurkat T-cell lysate to the GST-PAK1-GBD on the sensing surface. Hence, the maximum voltage sensitivity of the AuNis HEMT biosensor was achieved for about 33.00% at 3 × 10⁻⁷ g/mL Jurkat T-cell lysate concentration, as shown in Fig. 7f.

### Limit of detection and dissociation constant

**Fig. 8:**
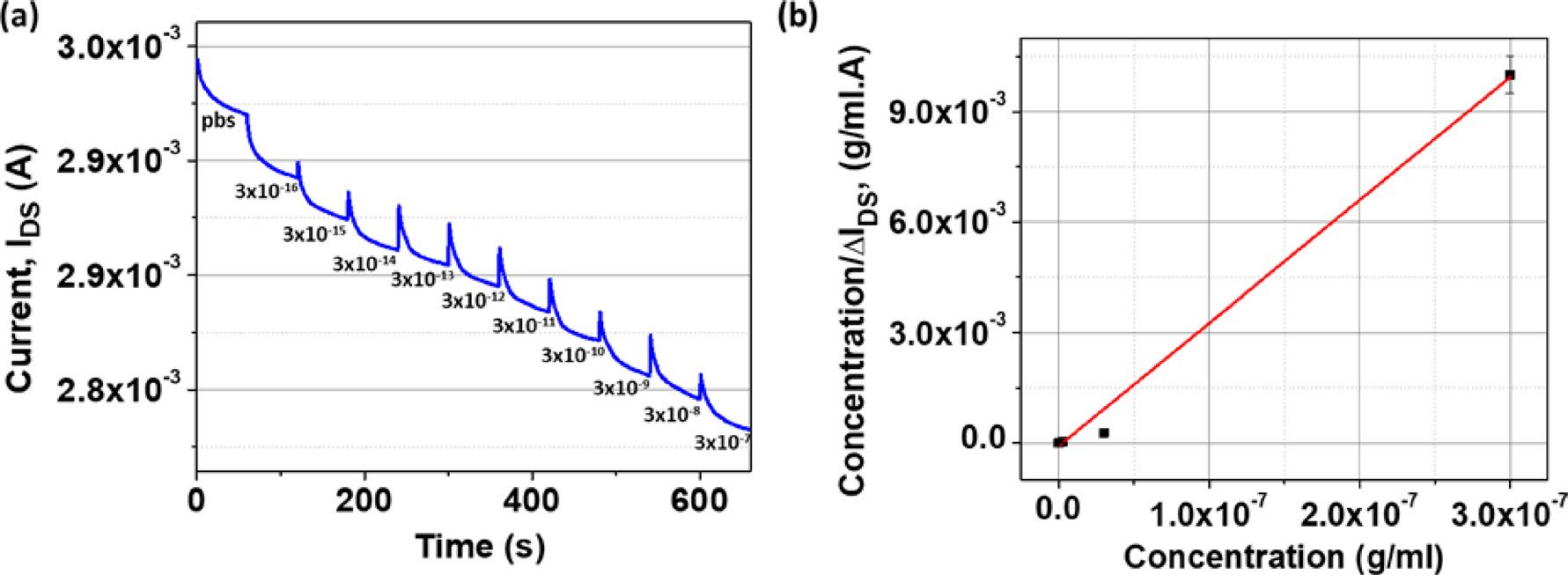
The graph of (**a**) real-time response of drain current (I_DS_) in varying concentrations of Jurkat T-cell lysate and (**b**) [Ag]/ΔI_DS_ versus [Ag] to extract dissociation constant (K_D_).

To further analyze the performance of the AuNis HEMT biosensor, the time-dependent response of the drain current (I_DS_) was monitored in response to varying concentrations of Jurkat T-cell lysate, as illustrated in Fig. 8a. It is noted that in Fig. 8a, the baseline had not stabilized before the next sample concentration was introduced, therefore, the response time and current changes cannot be determined. The AuNis HEMT biosensor exhibited a broad dynamic detection range from 3 × 10⁻^16^ g/mL to 3 × 10⁻⁷ g/mL, with a limit of detection (LOD) as low as 3 × 10⁻^16^ g/mL. Additionally, the LOD calculated using the 3σ method was 3.64 × 10⁻^15^ g/mL, approximately one order of magnitude higher than the experimental value, thereby supporting and validating the reliability of the experimental detection approach. The Table 1 below presents a comparative analysis of the sensing performance between the AuNis HEMT biosensor developed in this study and previously reported HEMT-based biosensors utilizing unannealed Au thin films for cancer-related biomarker detection. The AuNis HEMT demonstrates a markedly lower LOD compared to the unannealed Au thin film biosensor, highlighting the effectiveness of the AuNis structure as a highly functional sensing membrane. Such outstanding performance is largely attributed to the AuNis nanostructured surface, which offers a high surface-area-to-volume ratio and abundant active binding sites, thereby enhancing biomolecular capture efficiency. These findings position the AuNis HEMT biosensor as a highly sensitive and reliable platform for biomolecular diagnostics^43,44^.

**Table 1:** The comparison analysis of the sensing performance between unannealed Au thin film and AuNis HEMT-based biosensor.

Types of cancer	Sensing membrane	Linear range of concentration	Limit of detection	Refs.
Breast cancer	Au film	0.25–16.7 µg/mL	0.25 µg/mL	^46^
Breast cancer	Au film	4–131 µg/mL	4 µg/mL	^47^
Lung cancer	Au film	1 pM–500 pM	1 pM	^48^
Acute lymphoblastic leukemia cancer	AuNis	0.3 fg/mL–0.3 µg/mL	0.3 fg/mL	This work

Then, the chemical interaction between the ligand (small Rho GTPases from Jurkat T-cell lysate) and the receptor (GST-PAK1-GBD) in bulk solution can be described by the following equilibrium reaction:

2 $${\text{[Ab] + [Ag]}} \leftrightarrow {\text{[Ab - Ag]}}$$

where [Ab] denotes the concentration of GST-PAK1-GBD immobilized on the sensing surface, [Ag] represents the concentration of Jurkat T-cell lysate, and [Ab–Ag] is the concentration of the resulting receptor–ligand complex. The binding affinity between ligand and receptor for protein–protein interaction can be quantified using the dissociation constant (K_D_), based on the Langmuir adsorption isotherm for a one binding site model^45^. The change in the drain current (ΔI_DS_) in response to varying Jurkat T-cell lysate concentrations was used to estimate K_D_ using the following rearranged form of the Langmuir equation:

3 $$\frac{{\text{[Ag]}}}{{\Delta {\text{I}}_{{{\text{DS}}}} }}{ = }\frac{{\text{[Ag]}}}{{\Delta {\text{I}}_{{{\text{DSmax}}}} }}{ + }\frac{{{\text{K}}_{{\text{D}}} }}{{\Delta {\text{I}}_{{{\text{DSmax}}}} }}$$

Here, ΔI_DS_ is the current change at a given concentration of Jurkat T-cell lysate, and ΔI_DSmax_ is the maximum observed current shift. By plotting [Ag]/ΔI_DS_ versus [Ag], a linear regression was obtained, as shown in Fig. 8b. The slope and y-intercept of the linear fit correspond to 1/ΔI_DSmax_ and K_D_/ΔI_DSmax_, respectively. The fitted line yielded a high correlation coefficient (R^2^ ≈ 0.99), validating the one-site binding model. The extracted maximum current change (ΔI_DSmax_) from the Langmuir analysis was approximately 0.53 mA, consistent with earlier experimental results. The dissociation constant (K_D_) was calculated to be 5.20 × 10⁻^10^ g/mL, which is among the lowest reported values for HEMT-based biosensors for one binding site compared to previous studies^45^. This low K_D_ value suggests efficient immobilization of GST-PAK1-GBD on the AuNis sensing surface, and a strong affinity binding toward small Rho GTPases from the Jurkat T-cell lysate. In addition, the dissociation constant (K_D_) also provides insight into the number of available binding sites on the receptor, which is closely related to the biosensor’s limit of detection. However, the extracted K_D_ value does not align with the experimentally observed LOD, indicating that the data cannot be adequately fitted to a one-site binding model. Therefore, it can be concluded that the GST-PAK1-GBD (residues 56–272) receptor clearly has more than one binding site.

### Reproducibility and regeneration

**Fig. 9:**
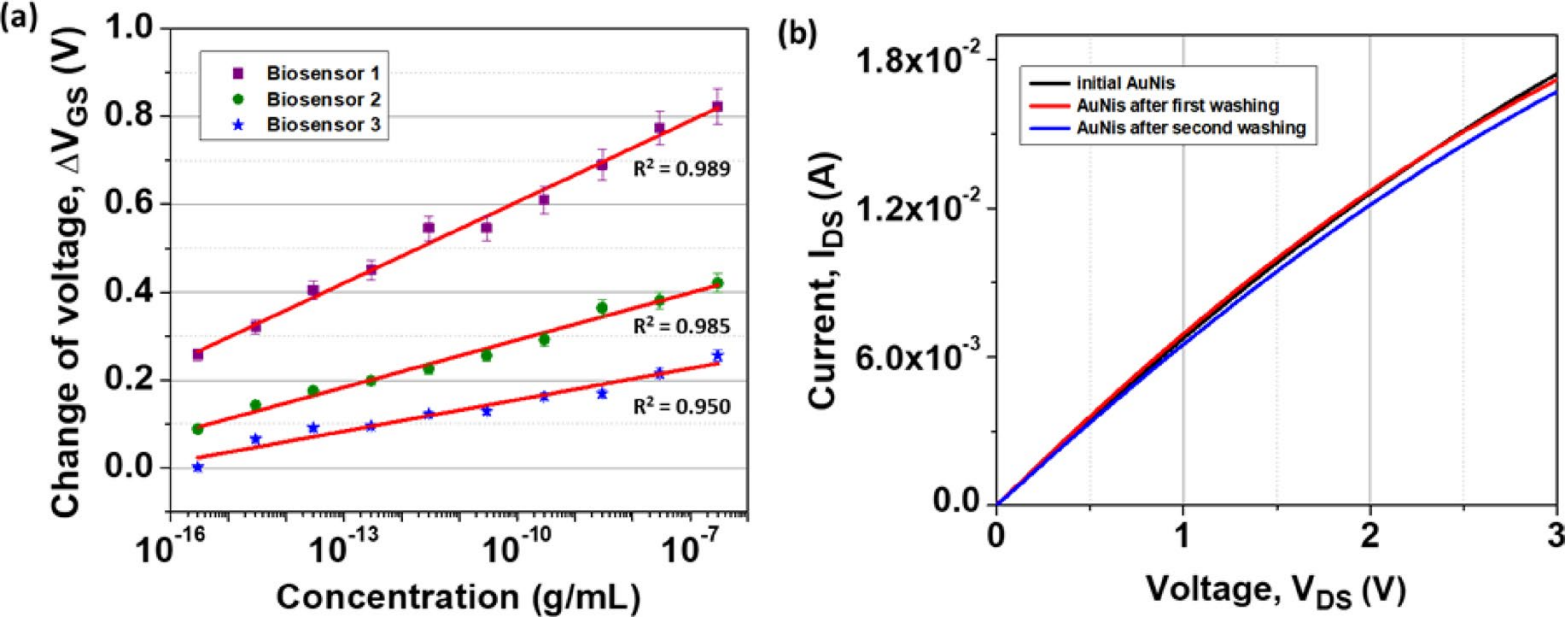
The graph of (**a**) linearity of ΔV_GS_ and the log of Jurkat T-cell lysate concentration for three AuNis HEMT biosensors and (**b**) the I_DS_ versus V_DS_ curves of the AuNis HEMT sensor for the initial state, after one washing cycle, and after two washing cycles.

The reproducibility of biosensors remains a major hurdle in the development reliable HEMT biosensors^49^. To validate the reproducibility of the biosensor, three biosensors were fabricated and measured under the same conditions. Figure 9a displays the linear relation of ΔV_GS_ and the log of Jurkat T-cell lysate concentration, representing R^2^ values of approximately 0.989, 0.985, and 0.950 for biosensor 1, biosensor 2, and biosensor 3, respectively. The incorporation of AuNis onto the HEMT biosensor demonstrated good reproducibility across three independent biosensors over a wide range of Jurkat T-cell lysate concentrations. The three biosensors exhibited a coefficient of correlation (R^2^) equal to or greater than 0.950 with a relative standard deviation (RSD) of 2.17%. This low RSD indicates minimal variation in results, suggesting excellent consistency in performance compared to a previous study of the HEMT biosensor with 4.4% RSD^50^. These findings highlight the capability of the AuNis HEMT biosensor to deliver reliable measurement outcomes, attributed to the uniform distribution and growth of the nanoislands across the sensing surface surpassing the significant challenge of the variability in the density of AuNPs fabricated on the HEMT surface^51^.

Regeneration is a critical process that restores the sensing surface to its original state prior to analyte interaction, thereby enabling repeated use and maintaining the biosensor’s performance. Figure 9b presents the output curves (I_DS_ versus V_DS_) with a constant V_GS_ of -1 V measured for the AuNis HEMT biosensor in three conditions: the initial state, after one washing cycle, and after two washing cycles. The drain current of the AuNis HEMT biosensor changed by approximately 1.39% with 98.61% restoration after one washing cycle, relative to its initial drain current at V_DS_ = 3 V. Achieving over 98% restoration of the initial current response after nearly one year represents a significant advancement, especially considering that previous studies reported similar recovery only up to 15 days^50^. Hence, this negligible drain current variation with high recovery efficiency highlights the sensor’s excellent regeneration capability, enabling reuse without significant performance degradation. Although a slight current change of approximately 4.13% with 95.87% restoration was observed in the AuNis HEMT biosensor after two washing cycles relative to its initial value, the sensor still demonstrated reliable operation, indicating its ability to function under harsh conditions and repeated regeneration steps. The biosensor’s ability to regenerate after multiple uses opens up new possibilities for its use in clinical settings, where cost-effectiveness and reusability are crucial. This capability enables the AuNis HEMT biosensor to address key limitations of conventional AlGaN/GaN biosensors, which degrade after single-use applications^52^.

## Conclusions

This study introduces AuNis as a gold nanostructure on an AlGaN/GaN HEMT sensor for biosensing applications for the first time. The use of an ultrathin Au film and a simple deposition process provides a cost-effective fabrication strategy. The AuNis HEMT sensor demonstrated excellent pH sensitivity and stability, validating its feasibility as a sensing membrane. Furthermore, the AuNis HEMT biosensor was successfully applied for the detection of small Rho GTPases in Jurkat T-cell lysate, exhibiting high current and voltage sensitivities of approximately 9.10% and 33.00%, respectively, at a concentration of 3 × 10⁻⁷ g/mL. The enhanced surface binding capability of AuNis contributed to a broad linear detection range (3 × 10⁻^16^ to 3 × 10⁻⁷ g/mL) and an ultra-low LOD of 3 × 10⁻^16^ g/mL. The biosensor also demonstrated excellent reproducibility, with an R^2^ equal to or exceeding 0.950, indicating uniform and consistent nanoisland distribution across the sensing surface. Moreover, over 98% signal recovery after rigorous washing steps further demonstrates the robustness and regeneration capability of the biosensor. With its high sensitivity, reproducibility, and robust surface characteristics, the AuNis-based AlGaN/GaN HEMT biosensor presents significant potential for broader biosensing applications.

## Data Availability

The datasets generated during and/or analysed during the current study are available from the corresponding author on reasonable request.
